# Thermoring basis for the proton-driven heat activation of a cation-selective channel in myriapods

**DOI:** 10.1038/s41598-025-31732-5

**Published:** 2025-12-15

**Authors:** Guangyu Wang

**Affiliations:** 1 Department of Physiology and Membrane Biology, University of California School of Medicine, Davis, CA 95616 USA; 2 Department of Drug Research and Development, Institute of Biophysical Medico-Chemistry, Reno, NV 89523 USA

**Keywords:** Biochemistry, Biophysics

## Abstract

Thermo-gated ion channels undergo significant time-dependent structural rearrangements for activation. Although the tertiary noncovalent interaction networks can be constrained as thermorings to explain specific temperature thresholds and sensitivity, the timing of critical intra-subunit and inter-subunit noncovalent interactions unfolding remains poorly understood. Here the tertiary noncovalent interaction networks of the broad-range thermal receptor 1 (BRTNaC1) in myriapods were quantified by the thermoring model to estimate thermal thresholds and structural unfolding sequences. The results showed that the theoretical threshold of the weakest intra-subunit noncovalent interaction in the resting closed state of the D217N/E218Q mutant at low temperature matched the experimental activation threshold. Further, the strong inter-subunit swapping interactions at H352, although weakened before strong acid activation of the native channel below pH 4.5, were absent before weak acid-induced heat activation of the mutant at pH 7. Therefore, the different unfolding sequence from strong inter-subunit interactions to the weakest intra-subunit bridges may be required for proton-driven heat activation of BRTNaC1.

**Supplementary Information:**

The online version contains supplementary material available at 10.1038/s41598-025-31732-5.

The timing of specific local unfolding is very important for activating thermo-gated ion channels. Protein folding usually advances from primary to secondary, then to tertiary, and finally to quaternary structures. However, multiple studies indicate that the heat activation of polymodal homotetrameric thermosensitive transient receptor potential vanilloid 1–4 (TRPV1-4) channels begins with the local specific unfolding of the tertiary structure and culminates in the quaternary structure.

First, in TRPV1, because protonation of E600 at pH 5.5 directly disrupts the external swapping R455-E600’ salt bridge and leads to channel opening^1–4^, unfolding the weakest internal Y401-R499 π bridge in rat TRPV1 (rTRPV1) or the least-stable internal E406-K504 salt bridge in human TRPV1 (hTRPV1) along the phosphatidylinositol (PI)-dependent minimal gating pathway above 41 °C before disrupting the swapping salt bridge is required to initiate heat activation^5–7^.

Second, in TRPV3, since disrupting the internal swapping K169-E751’ bridge with the K169A mutation directly opens human TRPV3 (hTRPV3) below room temperature^8^, unfolding the weakest K614-N647 H-bond along the phosphatidylcholine (PC)-dependent minimal gating pathway before disconnecting the highly conserved inter-subunit K169-E751’ salt bridge is also necessary to initiate heat activation of reduced TRPV3 above 50 °C^9–12^.

Finally, in TRPV2, given that a weak acid HoAC disrupts the intersubunit H521-R539’ cation-π interaction and opens rat TRPV2 (rTRPV2) at pH 5 and room temperature^13^, unfolding the weakest R369-D469 and L555-Y590 bridges along the phosphatidylethanolamine (PE)-dependent gating pathway of rat TRPV2 (rTRPV2) before disrupting the swapping π bridge is needed for initial heat activation above 48–53 °C or cold activation at 4 °C^14–18^. These findings are consistent with a previous report indicating that a similar swapping Y602-R616’ π interaction must be disrupted for final TRPV4 opening^19^. Taken together, unfolding the weakest intra-subunit tertiary noncovalent bridge is enough to open TRPV1-4 channels above specific thresholds.

However, recent studies on a broad-range thermal receptor 1 (BRTNaC1) in centipede antennae indicated that protonation below pH 6.5 switches on its high thermal sensitivity^20^. Since its temperature-dependent cryogenic electron microscopy (cryo-EM) structures have been available at various pH values^21^, it is necessary to test a hypothesis that the local specific unfolding sequence from tertiary to quaternary structures as found in thermo-gated TRPV1-4 channels is reversed in BRTNaC1.

To this end, the constrained tertiary noncovalent interaction networks or thermoring structures of BRTNaC1 with or without the D217N/E218Q mutations at pH7/8 and 4/40 °C were analyzed using a highly-sensitive grid thermodynamic model that has been recently developed and validated^6, 1217,18,22–29^. In this model, paired protein residues and relevant tertiary noncovalent interactions along a single polypeptide chain are represented as arrowed nodes and edges in a systematic grid-like mesh network, respectively. While any interaction forms a direct path, a nearby polypeptide segment with or without other interactions can pave the shortest reverse path so that the shortest round path can be defined as a grid with a path length as its size. As the temperature melting threshold (T_m,th_) for unfolding any interaction is determined not only by the interaction intensity but also by the grid size, the constrained grid is also called as a thermo-sensitive ring or thermo-ring. In this way, when all the tertiary interactions are labeled with unique sizes, the weakest interaction can typically be found in the biggest thermoring, predicting the protein’s melting threshold (T_m,th_). In addition, the ratio of total grid sizes to total tertiary interactions defines systematic thermal instability (T). Finally, the structural thermosensitivity (Ω_10_) is defined and calculated as the thermo-evoked change in the total chemical potential of grids during channel activation along the single polypeptide chain or the defined gating pathway, in response to a thermo-evoked change in total enthalpy from the noncovalent interactions. Once Ω_10_ matches the functional thermosensitivity (Q_10_), the origin of temperature sensitivity can be traced.

The results showed that disrupting the weakest noncovalent interaction in the pore domain along the single polypeptide of BRTNaC1 is enough to open the D217N/E218Q channel with matched temperature threshold and sensitivity. Further study revealed that the strong intersubunit π–π interactions among three H352 residues and three pairs of F350-H352’ must be disrupted before the weakest intra-subunit bridges in the pore domain during proton-driven heat activation. A potential activation pathway initiated by the protonation of H352 below pH 6.5 is also proposed and discussed.

## Results

### Strong swapping π interactions at H352 of the WT channel above pH 7 and below 52 °C

**Fig. 1:**
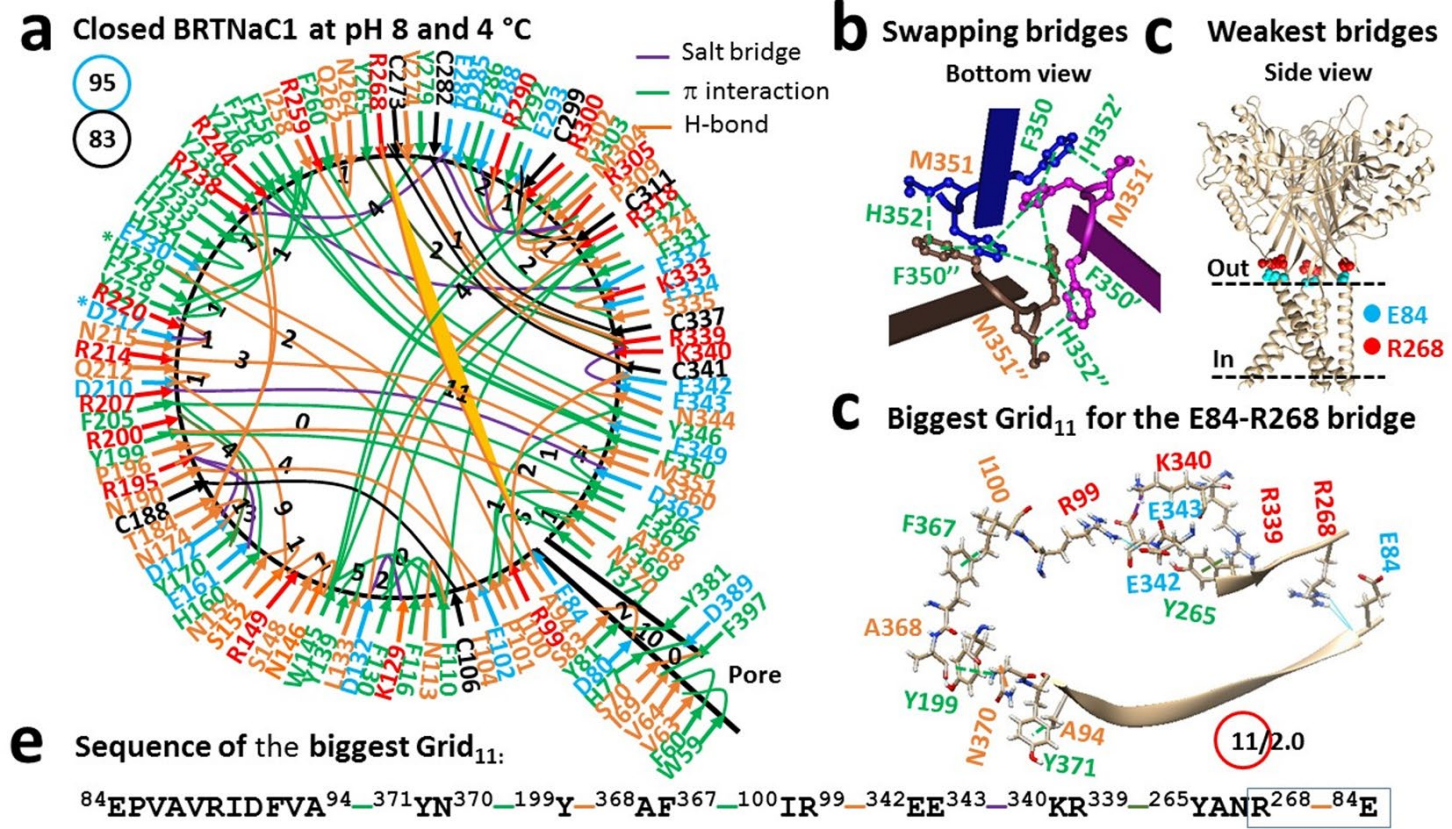
Thermoring structures of closed BRTNaC1 at pH 8 and 4 °C. (**a**) The grid-like noncovalently interacting mesh network along the gating pathway of closed BRTNaC1 based on cryo-EM data of a single subunit at pH 8 and 4 °C (PDB ID: 8YMR). Along the gating pathway from R53 to F414, in addition to black disulfide bonds, salt bridges, π interactions, and H-bonds between paired amino acid side chains are denoted in purple, green, and orange, respectively. The specific grid sizes necessary to regulate the least-stable noncovalent interactions in the grids are indicated with black numbers. The weakest E84-R268 bridge in the biggest Grid_11_ is highlighted in orange. The total grid sizes and the total grid size-controlled noncovalent interactions along the gating pathway from R53 to F414 are displayed in cyan and black circles, respectively. (**b**) The swapping interactions at H352. (**c**) The location of the weakest E84-R268 bridges. (**d**) The structure of the biggest Grid_11_ with an 11-residue size to regulate the E84-R268 bridge. (**e**) The sequences of the biggest Grid_11_ to control the highlighted E84-R268 bridge in the blue box.

Given that BRTNaC1 is activated below pH 6.5 and a histidine residue has an average pKa value of 6.0^20,30^, the primary search for interactions with His started from the wild type (WT) channel at pH 8 (PDB:8YMR). When a total of 83 tertiary noncovalent interactions formed a systematic grid-like mesh network, a total of 95 grid sizes resulted in a systematic thermal instability (T_i_) of 1.14 (Fig. 1a, Tables 1 and S1). Notably, while T104-H229-Y227, H231-H232, H233-H235/Y279 bridges in the extracellular domain were controlled by smaller grids with a size range from 0 to 4, the H77-D389 π interaction, together with the Y81-Y381 bridge, was governed by Grid_10_ in the pore domain (Fig. 1a). In contrast, when strong swapping π interactions among three H352 residues and between F350 and M351’/H352’ generated the smallest Grid_0_ (Fig. 1b), the calculated melting threhold (T_m,th_) was at least 120 °C.

**Table 1:** Comparison of acid- and heat-induced thermoring structural changes in BRTNaC1 along the gating pathway from R53 to F414. The comparative parameters are highlighted in bold.

PDB ID	8YMR	8YMS	8YMU	8YMX	8YMW
Construct	WT			D217N/E218Q	
Sampling temperature, °C	4			40	4
Sampling pH	8	4		7	7
Gating state	Closed	Closed	Open	Open	Closed
# of the biggest Grids	Grid_11_	Grid_9_	Grid_7’_	Grid_7_	Grid_14_
grid size (s)	11	9	7	7	14
The weakest noncovalent bridge	E84-R268	H77-D389	K122-E218	R244-E332	I69-F397
# of basic H-bonds (n) in stability equivalent to the weakest noncovalent bridge	2.0	1.0	1.0	1.0	1.0
Total non-covalent interactions (N)	83	76	75	69	76
Total grid sizes (S), a.a	95	95	97	76	94
Systemic thermal instability (Ti)	1.14	1.25	1.29	1.10	1.24
Calculated T_m,th_ °C	**52**	**46**	**50**	**50**	**36**
Measured threshold T_th_, °C	**50**			**48**	**33**
Calculated T_m,th_ °C at E = 0.5 kcal/mol				5.2	
Calculated Ω_10_, mean at E = 1.0 kcal/mol		**-1**		**11.2**	
Calculated T_m,th_ °C at E = 3 kcal/mol				37.5	
Measured Q_10_				**10.8**	
Refs for T_th_ and Q_10_	(20)			(20)	(20)

Furthermore, the biggest Grid_11_ along the single polypeptide chain was found to govern the weakest E84-R268 H-bond near the extracellular gate through a thermoring from E84 to A94, Y371, N370, Y199, A368, F367, I100, R99, E342, E343, K340, R339, Y265, R268 and back to E84 (Fig. 1a, c–e). Since this bridge was energetically equivalent to 2.0 basic H-bonds (2 kcal/mol), the calculated T_m,th_ was 52 °C (Table 1). Therefore, both T_i_ and T_m,th_ suggest that the WT channel is stable above pH 7 and below 52 °C^20^.

### Disrupting intersubunit interactions at H352 are required for the matched heat threshold

Given that the heat activation of the mutant D217N/E218Q exhibited a similar threshold of 33 °C and thermosensitivity of 11 as the WT channel at pH 6.1^20^, the double mutant similarly reflects the protonated state of the WT channel at pH 6.1. Therefore, it is intriguing to investigate whether the swapping interactions at H352 are disrupted prior to heat activation.

**Fig. 2:**
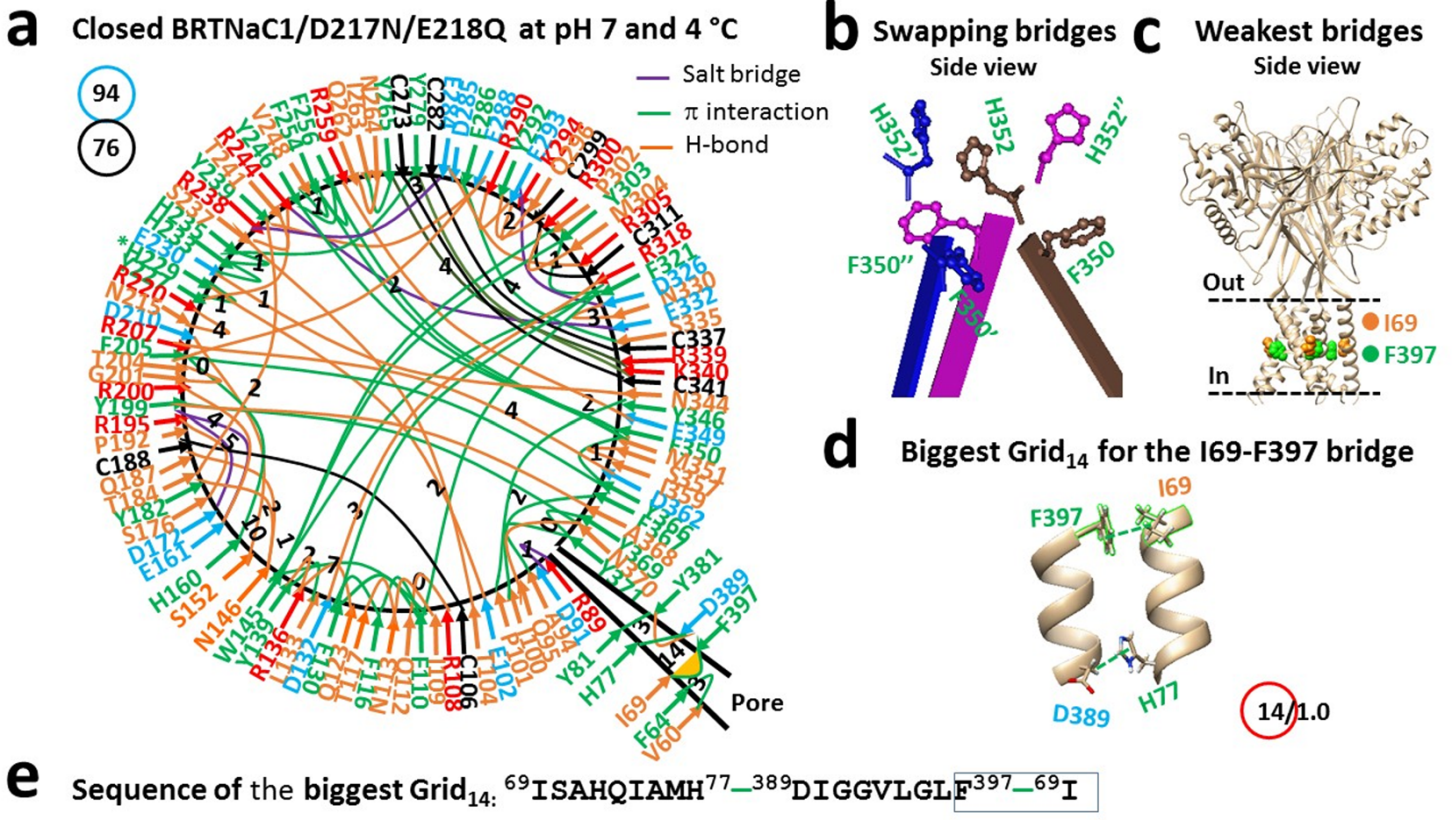
Thermoring structures of closed BRTNaC1/D217N/E218Q at pH 7 and 4 °C. (**a)** The grid-like noncovalently interacting mesh network along the gating pathway of closed BRTNaC1/D217N/E218Q based on cryo-EM data of a single subunit at pH 7 and 4 °C (PDB ID: 8YMW). Along the gating pathway from R56 to V411, in addition to black disulfide bonds, salt bridges, π interactions, and H-bonds between paired amino acid side chains are denoted in purple, green, and orange, respectively. The specific grid sizes necessary to regulate the least-stable noncovalent interactions in the grids are indicated by black numbers. The weakest I69-F397 bridge in the biggest Grid_14_ is highlighted in orange. The total grid sizes and the total grid size-controlled noncovalent interactions along the gating pathway from R56 to V411 are displayed in cyan and black circles, respectively. (**b**) The disrupted swapping interactions at H352. (c**)** The location of the weakest I69-F397 bridges. (**d**) The structure of the biggest Grid_14_ with a 14-residue size to regulate the I69-F397 bridge. (**e**) The sequences of the biggest Grid_14_ to control the highlighted I69-F397 bridge in the blue box.

Along the single structured polypeptide chain of the closed mutant D217N/E218Q from R56 to V411, seventy-six tertiary noncovalent interactions were identified at pH 7 and 4 °C. These interactions included 6 salt bridge, 34 H-bonds and 36 π interactions (Fig. 2a, Table S2). When these interactions formed a systematic grid-like mesh network, a total of grid sizes was 94 (Fig. 4a). Therefore, the systematic thermal instability (T_i_) was 1.24 (Table 1).

Notably, despite the H77-D389, Y227-H229, E230-H233-H235-Y279 π interactions (Fig. 4a), the strong swapping interactions at H352 are disrupted (Fig. 2b). Consequently, the biggest Grid_14_ was responsible for the weakest I69-F397 π bridge in the pore domain (Fig. 2a and c). It cycled from I69 to H77, D389, F397, and back to I69 (Fig. 2d,e). When the I69-F397 bridge was energetically equivalent to 1.0 basic H-bond (1 kcal/mol), the calculated melting temperature threshold (T_m,th_) was 36 °C. When a lower NaCl concentration (140 mM) is considered for the measurement, the predicted threshold based on 150 mM NaCl is similar to the experimental threshold of 33 °C of both the D217N/E218Q mutant at pH 7 and the WT channel at pH 6.1 (Table 1)^20,31–35^. Therefore, disrupting the swapping bridges at H352 is required to match the threshold for proton-driven heat activation below pH 6.5.

### Disrupting H352-H352’-H352’’ bridges is necessary for matched thermosensitivity

**Fig. 3:**
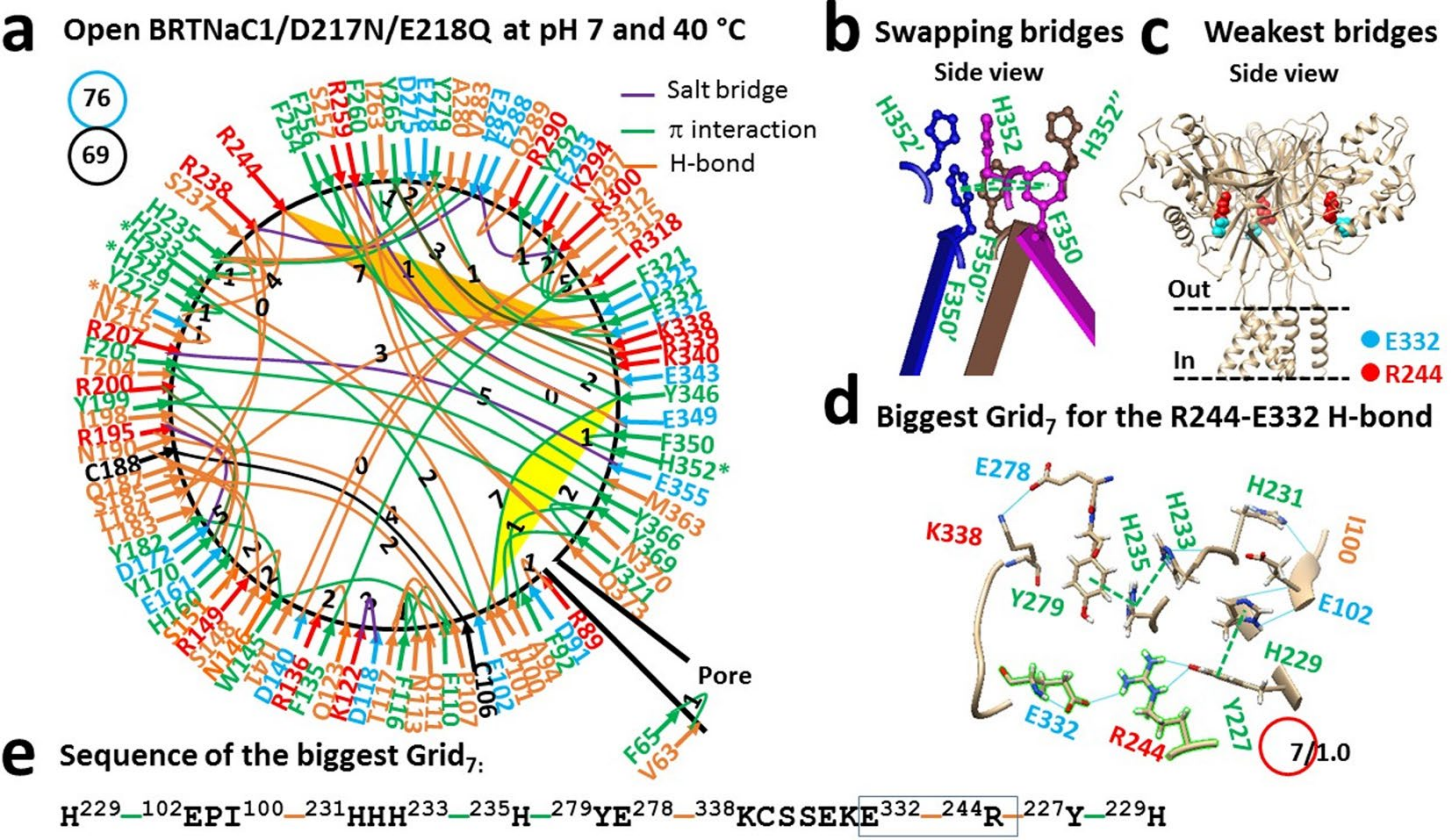
Thermoring structures of open BRTNaC1/D217N/E218Q at pH 7 and 40 °C. (**a**) The grid-like noncovalently interacting mesh network along the gating pathway of open BRTNaC1/D217N/E218Q based on cryo-EM data of a single subunit at pH 7 and 40 °C (PDB ID: 8YMX). Along the gating pathway from V63 to I400, in addition to a black disulfide bond, salt bridges, π interactions, and H-bonds between paired amino acid side chains are denoted in purple, green, and orange, respectively. The specific grid sizes necessary to regulate the least-stable noncovalent interactions in the grids are indicated with black numbers. The first weakest R244-E332 bridge in the biggest Grid_7_ is highlighted in orange while the second weakest P101-Y346 bridge is marked in yellow. The total grid sizes and the total grid size-controlled noncovalent interactions along the gating pathway from V63 to I400 are displayed in cyan and black circles, respectively. (**b**) The swapping interactions at H352. (**c**) The location of the first weakest R244-E332 bridges. (**d**) The structure of the biggest Grid_7_ with a 7-residue size to regulate the R244-E332 bridge. (**e**) The sequences of the biggest Grid_7_ to control the highlighted R244-E332 bridge in the blue box.

When the temperature increased to 40 °C to open the mutant channel, the weakest I69-F397 π bridge was disrupted as expected (Fig. 3a). Along with three swapping F350-F350’-F350’’ π interactions (Fig. 3b), the new weakest R244-E332 H-bond was born in the extracellular domain (Fig. 3a,c). It was controlled by the biggest Grid_7_ via a thermoring from H229 to E102, I100, H231, H233, H235, Y279, E278, K338, E332, R244, Y227 and back to H229 (Fig. 5c–e). Since this H-bond had the energy equivalent to 1.0 basic H-bond, the calculated T_m,th_ was 50 °C, which closely matched the measured maximal activity temperature of 48 °C after taking into account the lower NaCl concentration (140 mM) used for the measurement (Table 1)^20,31–35^.

Together with the change in the weakest bridge from I69-F397 to R244-E332, the total numbers of tertiary noncovalent interactions and grid sizes decreased from 76 to 69 and 94 to 76, respectively (Figs. 2a and 3a; Tables S2 and S3). As a result, the systematic thermal instability (T_i_) decreased from 1.24 to 1.10 (Table 1). Assuming the average energy of the noncovalent interactions was 1 kcal/mol, the calculated mean structural thermosensitivity (Ω_10_) was 11.2, which closely matched the measured functional thermosensitivity (Q_10_) of 10.8 (Table 1)^20^. Accordingly, the broken H352-H352’-H352’’ swapping bridges are also required for matched temperature sensitivity. With four broken bridges reducing 19 grid sizes in the pore domain during channel activation by heat, the conformational change in the pore domain contributed the most to the higher temperature sensitivity.

### Disrupting the swapping bridges at H352 is necessary for temperature-independent acid activation

**Fig. 4:**
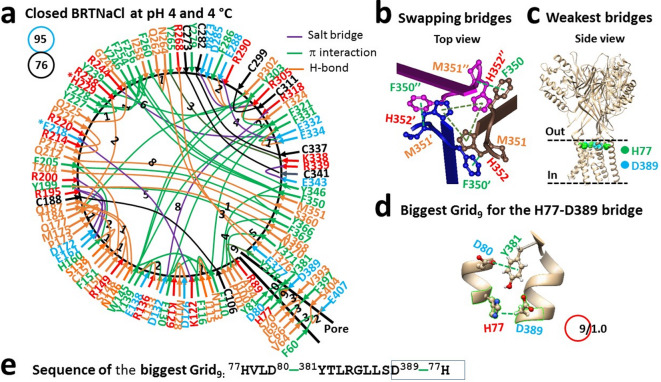
Thermoring structures of closed BRTNaC1 at pH 4 and 4 °C. (**a**) The grid-like noncovalently interacting mesh network along the gating pathway of closed BRTNaC1 based on cryo-EM data of a single subunit at pH 4 and 4 °C (PDB ID: 8YMS). Along the gating pathway from R53 to F414, in addition to black disulfide bonds, salt bridges, π interactions, and H-bonds between paired amino acid side chains are denoted in purple, green, and orange, respectively. The specific grid sizes necessary to regulate the least-stable noncovalent interactions in the grids are indicated with black numbers. The weakest H77-D389 bridge in the biggest Grid_9_ is highlighted in orange. The total grid sizes and the total grid size-controlled noncovalent interactions along the gating pathway from R56 to V411 are displayed in cyan and black circles, respectively. (**b**) The swapping interactions at H352. (**c**) The location of the weakest H77-D389 bridges. (**d**) The structure of the biggest Grid_9_ with a 9-residue size to regulate the H77-D389 bridge. (**e**) The sequences of the biggest Grid_9_ to control the highlighted H77-D389 bridge in the blue box.

If disrupting the swapping bridges at H352 is required for proton-driven heat activation, it should also be needed for acid activation. When the pH decreases from 8 to 4, despite some noncovalent interactions such as intra-subunit H77-D389 and Y227-H229 bridges, the inter-subunit H352-H352’-H352’’ bridges were weakened along with the F350-M351’ bridges (Fig. 4a,b). Of special interest, when the weakest E84-R268 bridge at pH 8 was disrupted by strong acidification at pH 4, the H77-D389 bridge in the pore domain became the least-stable (Fig. 4c). It was controlled by the biggest Grid_9_ via a thermoring from H77 to D80, Y381, D389, and back to H77 (Fig. 4d,e). When this bridge was energetically equivalent to 1.0 basic H-bond (1 kcal/mol), the T_m,th_ was calculated as 46 °C (Table 1). Meanwhile, following a decrease in the total noncovalent interactions from 83 to 76 , the systematic thermal instability (Ti) increased from 1.14 to 1.25 (Fig. 4a, Tables 1 and S4). Hence, strong acidification destabilized the WT channel along with the weak swapping interactions at H352.

**Fig. 5:**
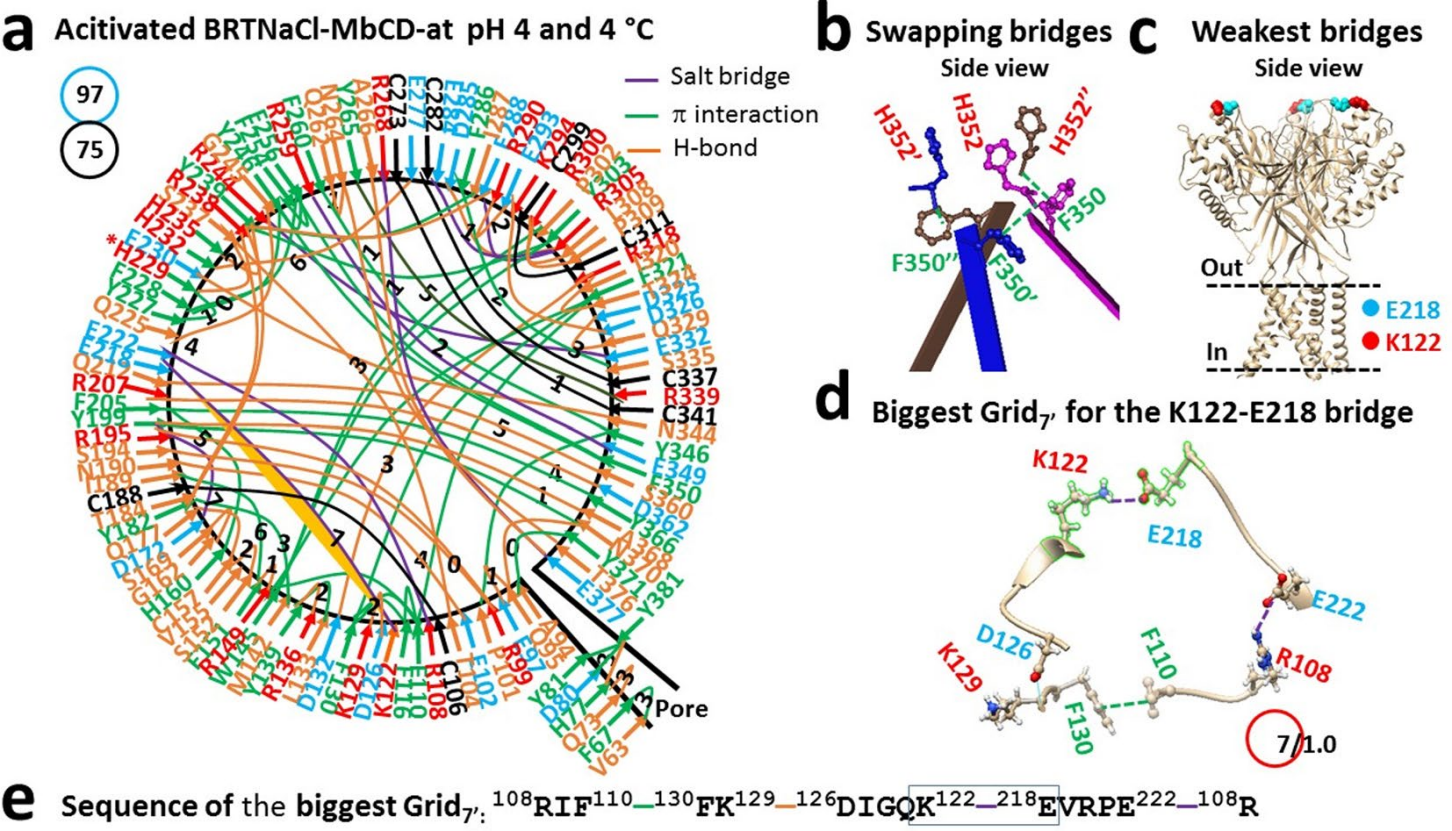
Thermoring structures of open BRTNaC1 at pH 4 and 4 °C. (**a**) The grid-like noncovalently interacting mesh network along the gating pathway of open BRTNaC1 based on cryo-EM data of a single subunit at pH 4 and 4 °C (PDB ID: 8YMU). Along the gating pathway from R53 to F414, in addition to a black disulfide bond, salt bridges, π interactions, and H-bonds between paired amino acid side chains are denoted in purple, green, and orange, respectively. The specific grid sizes necessary to regulate the least-stable noncovalent interactions in the grids are indicated with black numbers. The weakest K122-E218 bridge in the biggest Grid_7’_ is highlighted in orange. The total grid sizes and the total grid size-controlled noncovalent interactions along the gating pathway from R53 to F414 are displayed in cyan and black circles, respectively. (**b**) The swapping interactions at H352. (**c**) The location of the weakest K122-E218 bridges. (**d**) The structure of the biggest Grid_7’_ with a 7-residue size to regulate the K122-E218 bridge. (**e**) The sequences of the biggest Grid_7’_ to control the highlighted K122-E218 bridge in the blue box.

When the weakest H77-D389 bridges were disrupted by protonation of D389 at pH 4^21^, the channel was activated with the H77-Q73, H232-E277 and H235-R238 H-bonds (Fig. 5a). Notably, along with the swapping H352-F350’ π interactions (Fig. 5b), the weakest K122-R218 salt bridge in the extracellular domain was governed by the biggest Grid_7’_ through a thermoring from R108 to F110, F130, K129, D126, K122, E218, E222, and back to R108 (Fig. 5c–e). With 1.0 equivalent basic H-bond, this salt bridge had a T_m,th_ of 50 °C (Table 1).

On the other hand, owing to a subtle increase in the total of noncovalent interactions and grid sizes from 76 to 75 and 95 to 97 during strong acid activation, respectively (Figs. 4a and 5a), the systematic thermal instability (T_i_) was 1.29 and the structural thermosensitivity (Ω_10_) was -1 (Table 1). Therefore, the strong acid activation was temperature-independent along with the broken swapping interactions at H352.

## Discussion

The temperature-dependent gating of a thermosensitve ion channel involves a complex conformational change at both tertiary and quaternary structural levels. Recent studies have shown that disrupting the weakest tertiary noncovalent link is enough to separate an inter-subunit bridge for the thermo-gated activation of TRPV1-4 channels above specific thresholds^1–19^. However, this study found a different sequence. The inter-subunit π bridges at H352 were disrupted before the weakest bridges below pH 6.5 for heat activation with matched temperature threshold and sensitivity.

### Role of the D217N/E218Q mutation in mimicking the protonated WT channel at pH 6.1

Based on the thermoring model and assuming 1 kcal/mol as the average energy intensity of a noncovalent bridge^36^, three calculated parameters such as T_m,th_, T_i_, and Ω_10_ have been used to uncover the temperature-dependent gating pathways of TRPV1-4 channels once they align well with the experimental data^6,12,17,18,26^. For example, unfolding the weakest K614-N647 H-bond in the pore domain of reduced mouse TRPV3 (mTRPV3) without the C612-C619 disulfide bond above the matched threshold of 52 °C triggers the initial heat activation with the higher thermoensitivity of 21 (Ω_10_ = Q_10_). In contrast, unfolding of the least-stable R416-D519 salt bridge at the interface between the pre-S1 domain and the voltage sensor-like domain of oxidized mTRPV3 with the C612-C619 disulfide bond above the matched 40 °C stimulates the second heat activation with the lower thermosensitivity of 2.7 (Ω_10_ = Q_10_). Furthermore, when the stabler open oxidized state with the C612-C619 disulfide bond (T_i_ = 1.20) is shared by both closed states with lower and higher thresholds, these three states are enough to account for the use-dependent heat response^9–12,32^.

In this computational study, the calculated T_m,th_ values were all consistent with the experimental data. For example, the T_m,th_ values were 52 °C for closed WT at pH 8, 46 °C for closed WT at pH 4, 50 °C for open WT at pH 4, 36 °C for closed D217N/E218Q at pH 7, and 50 °C for open D217N/E218Q at pH 7 (Table 1)^20,21^. Moreover, the small calculated structural thermosensitivity between closed and open WT at pH 4 (Ω_10_ = -1) was in line with the temperature-independent strong acid activation^21^. In contrast, the predicted Ω_10_ of 11 between closed and open D217N/E218Q also aligned well with the experimental thermosensitivity (Q_10_ = 11). Finally, T_i_ values of 1.10–1.14 for the closed WT channel at pH 8 and open D217N/E218Q at pH 7 indicated stability, while the higher T_i_ of 1.24–1.25 for the WT channel at pH 4 and closed D217N/E218Q at pH 7 suggested potential instability (Table 1). Collectively, these parameters demonstrate that the D212N/E218Q mutant partially mimics the protonated state of the WT channel at pH 6.1. However, there are limitations. Firstly, the WT channel has a higher Q_10_ of 13 compared to the double mutant (Q_10_ = 11)^20^. Secondly, although the weakest H77-D389 or I69-F397 bridges were found in the pore domain of the closed WT channel at pH 4 or the closed mutant to initiate acid or heat activation (Figs. 2a, 3a, 4a, 5a), the D389A mutation does not open the channel as expected from pH 7 to pH 3.5^20,21^, possibly due to the mutation-induced inactivation at the D389-involved selectivity filter^21^. Thirdly, the C273-C341, C282-C337 and C299-C311 disulfide bonds in the closed mutant at 4 °C are broken in the open state at 40 °C (Figs. 2a and 3a). However, it is unknown if the same redox change occurs during weak-acid-induced heat activation of the WT channel. Lastly, this double mutant cannot define the initial protonation site of the WT channel at D217, E218 or H352 for the proton-driven heat activation.

### E218 is not the primary site of protonation for heat activation at pH 6.1

**Fig. 6:**
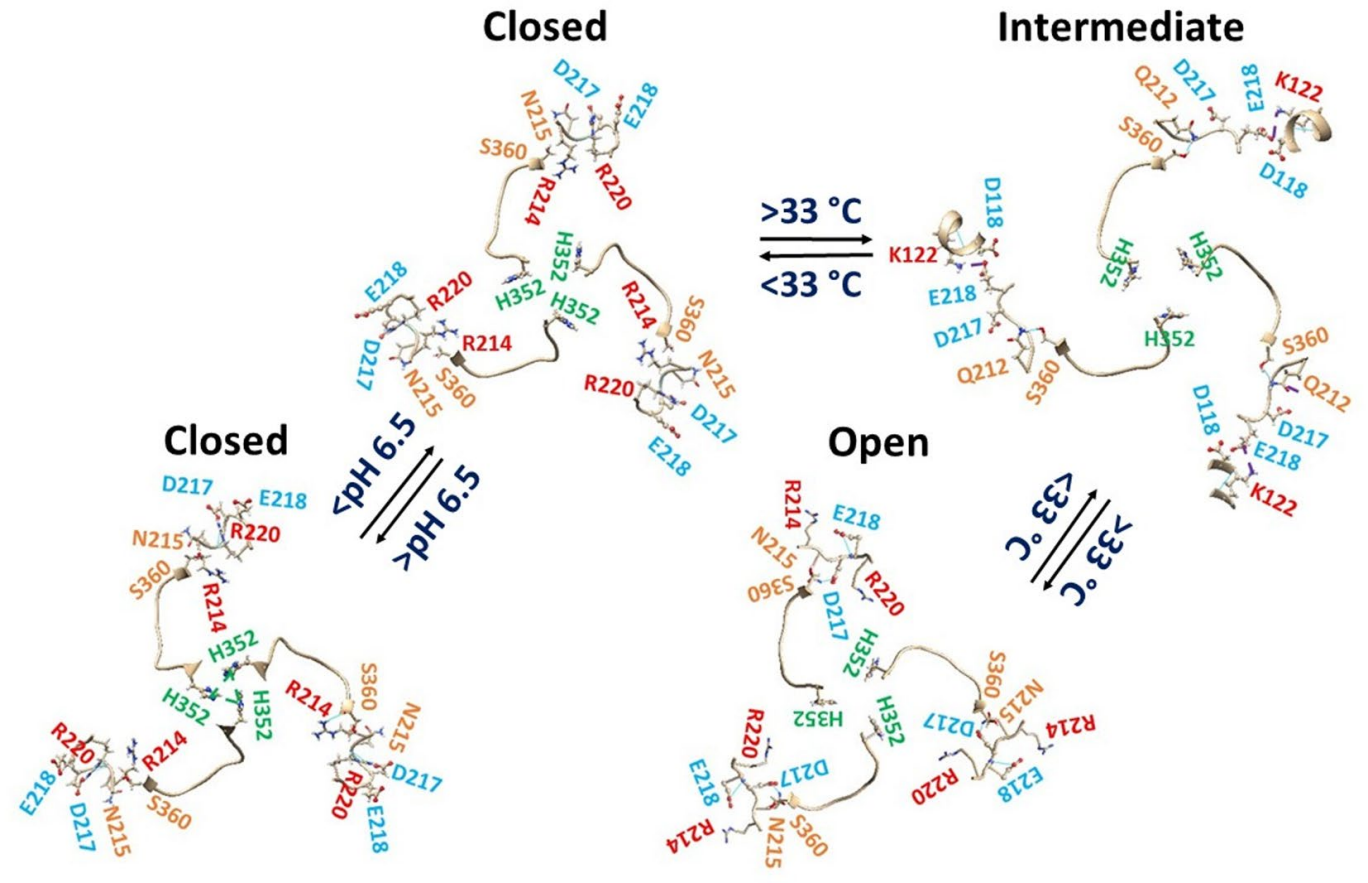
Tentative model on the proton-switched heat activation of BRTNaC1. Cryo-EM structures of closed BRTNaC1 at pH 8 and 4 °C (PDB ID: 8YMR), closed BRTNaC1/D217N/E218Q at pH 7 and 4 °C (PDB ID: 8YMW), activated BRTNaC1 at pH 4 and 4 °C (PDB ID: 8YMU), and open BRTNaC1/D217N/E218Q at pH 7 and 40 °C (PDB ID: 8YMX) were used to create this model. The strong swapping π interactions at H352 close the channel above pH 6.5. However, primary protonation of H352 below pH 6.5 disrupts these interactions, leading to a series of conformational rearrangements from Q212 to E218 via S360, resulting in channel opening above 33 °C.

Given that the E218D mutant is unresponsive to heat even above pH 4.8, it was proposed that E218 rather than D217 is a likely protonation site for heat activation at pH 6.1^20^. This study revealed that D217 formed a salt bridge with R220 in the WT channel at pH 8 (Fig. 1a). Therefore, only E218 was available for protonation. However, its pKa is about 4.25 for the side chain carboxyl group, which prevents E218 from being protonated at pH 6.1. Consequently, the heat activation induced by the D217N or E218Q or D217N/E218Q mutation at pH 7 may be due to an allosteric effect that disrupts the swapping π–π interactions among three H352 residues and three pairs of F350-H352’ in the trimeric BRTNaC1 channel after the D217-R220 salt bridges are broken (Fig. 6). Several similar cases were also reported in the F660S mutant of hTRPV1, the T633A or V538L mutant of rTRPV1, which suppresses proton activation but not capsaicin or heat activation^37,38^, possibly affecting the nearby swapping R455-E600’ salt bridge indirectly^5^. Further electrophysiological measurements or molecular dynamics simulations using the H352R/F mutations are necessary to clarify the role of D217N/E218Q mutant in mimicking the protonated wild-type channel below pH 6.5.

### Disrupting the swapping π bridges at H352 primes the heat activation

Recent structural data indicate that unfolding the weakest intra-subunit tertiary noncovalent interactions and then swapping R455-E600’, H521-R539’, K169-E751’ or Y602-R616’ bridges in the corresponding TRPV1, TRPV2, TRPV3 or TRPV4 channel is required for thermo-gated activation with significant thermosensitivity^5,11,13,15,17–19^. However, once the swapping of H352-H352’-H352’’ interactions, along with three pairs of F530-H532’ π interactions, formed the most stable triangular thermoring with a T_m,th_ higher than 120 °C, that thermoring could prevent heat activation even if the weakest tertiary I69-F397 bridge had been broken by the D389A mutation (Figs. 1, 2, 3)^21^. In line with this observation, similar swapping H352-H352’-H352’’ and F530-M531’ π interactions also closed the WT channel at pH 4 regardless of the separate D80-R384’ salt bridges^21^.

Notably, similar gating switches were also found in TRPV channels. For example, four H651 residues in rTRPV2 also form strong π–π interactions in the resting closed state at pH 7 but separate in the activated state in the presence of HoAC at pH 5^13^. Further, four swapping π–π interactions were also found at W583 of TRPV5 and W583A significantly increased Ca^2+^ influx and cell death^39,40^. Finally, the Y602C-R616C’ disulfide bond also locks TRPV4 in a closed state^19^. Therefore, the strong inter-subunit π bridges among three H352 residues and three pairs of F530-H532’ must be disrupted by acidification to minimize the energy barrier for the heat activation of BRTNaC1 below pH 6.5 above 33 °C (Figs. 1, 2, 3), as well as proton activation below pH 4.5 below 33 °C^21^.

### Primary protonation of H352 is required to break the swapping bridges at H352 for proton-driven heat activation

Unlike BRTNaC1, the acid-sensing ion channel (ASIC) in the same family is insensitive to temperature^21^. It has been observed that the H73A mutation in rat ASIC1a suppresses weak acid-induced activation^41^. Therefore, it is reasonable to assume that in the alkaline pH-induced closed state, the swapping H74-K77’ CH-π interactions, similar to the H521-R539’ cation-π interactions in rTRPV2^13^, coexist with not only the intrasubunit Y425-D433 H-bonds but also the strong intersubunit K373-E374’ H-bonds^42^, which resemble the swapping bridges at H352 of BRTNaC1 (Fig. 1b).

When the swapping H74-K77’ interactions are primarily disrupted upon exposure to protons, several pairs of negatively charged residues such as E80 and E417, E220 and D408, E239 and D346, and D238 and D350 become close enough to share a protonated water molecule or a proton via salt bridges or H-bonds. This collapsing of the acidic pocket disrupts Y425-D433 H-bonds and weakens swapping K373-E374’ bridges. As a result, three constricted D433 residues (equivalent to D389 in BRTNaC1) are released from the pore, allowing for channel opening^43,44^. However, the weak K373-E374’ swapping salt bridges are unstable unless stabilized by psalmotoxin (PcTx1) or MitTx (a snake toxin) in the open state^43,44^. Once these bridges are broken along with the disconnected intrasubunit Y425-D433 H-bonds, the three D433 residues merge together again to occlude the ion channel in the desensitized state^45^. A similar conformational change has been reported in human ASIC1a^46^. Therefore, protonation of a series of residues such as H74, E80, E417, E220, D408, D238, D346, E239 and D350 at pH 5.5 is required to weaken the swapping K373-E374’ bridges for ASIC1a opening. Furthermore, both E600 and D654 in TRPV1 are also close enough to share a proton or a protonated water molecule at pH 6, disconnecting the swapping E600-R455’ bridges for weak acid activation^3,4^.

However, the inter-subunit π bridges at H352, although weakened at pH 4 for strong acid activation (Fig. 4b), were broken with N217 and Q218 separated rather than H-bonded together in the closed state (Figs. 2a and 6). Therefore, it may be impossible for both D217 and E218 to share a proton or a protonated water molecule at pH 6.1. On the other hand, the E218D mutation decreases the threshold from pH 6.5 to pH 4.8 for heat activation above 33 °C^20^. Therefore, it is proposed that primary protonation of H352 below pH 6.5 disrupts the swapping bridges at H352, as well as the R214-S360 H-bond and the D217-R220 salt bridge (Fig. 6). When the channel is activated above 33 °C, S360 H-bonds with Q212 along with the K122-E218 salt bridge in an intermediate state based on the proton-activated state (PDB: 8YMU). However, this state is unstable (T_i_ = 1.29). Therefore, when it transitions to the stable heat-evoked open state (T_i_ = 1.10), the Q212-S360 and K122-E218 bridges are broken but N215 H-bonds with D217 via their side chains. For the E218D mutation, the short side chain is not enough to bridge with K122 in the intermediate. However, D218 may share a proton with nearby D118 below pH 4.8 for heat activation. For the E218Q mutation, a rotamer of the side chain can still H-bond with D118 even if it cannot form a salt bridge with K122 (Fig. 6). In this regard, although the intermediate has no effect on the Q_10_ of 11–13 (Table 1), it is still necessary for weak acid-induced heat activation to maintain consistent experimental data^20,21^. Further electrophysiological measurements or molecular dynamic simulations are necessary to examine this unique weak acid-induced heat activation pathway above 33 °C.

## Conclusion

The precise timing of specific intra-subunit and intersubunit noncovalent interactions is essential for the heat activation of thermosensitive ion channels but cryo-EM structures with adequate time resolution are currently unavailable. Here, comparative thermoring analyses of BRTNaC1 during acid and heat activations below pH 7 have shown that disrupting inter-subunit interactions at H352, while not necessary before strong acid activation, is required before weak acid-induced heat activation. Therefore, this study enhances our understanding of the unfolding sequence during the heat-evoked activation of thermo-gated ion channels.

## Methods

### Data mining resources

The full-length cryo-EM 3D structures of closed BRTNaC1 at pH 8 and 4 °C (PDB ID: 8YMR, model resolution = 3.09 Å), closed BRTNaC1/D217N/E218Q at pH 7 and 4 °C (PDB ID: 8YMW, model resolution = 3.2 Å), and open BRTNaC1/D217N/E218Q at pH 7 and 40 °C (PDB ID: 8YMX, model resolution = 3.27 Å) were analyzed to reveal the thermodynamic basis for weak acid-induced heat activation of BRTNaC1. The cryo-EM structures of closed and open BRTNaC1 at pH 4 and 4 °C were also used as controls (PDB ID: 8YMS and 8YMU, model resolution = 3.14 Å and 2.89 Å, respectively)^21^.

### Filtering tertiary noncovalent interactions

The stereo-selective and regio-selective inter-domain diagonal and intra-domain lateral tertiary noncovalent interactions along the gating pathway of BRTNaC1 from R53 to F414 were analyzed using UCSF Chimera. The interactions were filtered by the same strict and consistent standard as previously used and confirmed^6,12,17,18,22–29^. The examined noncovalent interactions included salt bridges, lone pair/CH/cation-π interactions and H-bonds between paired amino acid side chains. Specific cutoff distances and interaction angles to filter the different noncovalent interactions can be found in the online Supporting Information (Table S1, S2, S3, S4 and S5). It shoud be noted that momentary fluctuation-induced perturbations in tertiary noncovalent interactions during protein dynamics were not considered in this computational study.

### Mapping thermoring structures using a thermoring model

The study utilized the same grid thermodynamic model that was previously described and validated to map the systematic fluidic grid-like noncovalent interaction mesh network^6,12,17,18,22–29^. In this network, a topological grid was created with arrowed nodes representing amino acids and linked nodes representing noncovalent interactions along a single polypeptide chain. Graph theory and the Floyd–Warshall algorithm were employed to constrain the network as a thermoring structure with the grid size defined by the shortest round path length to control the least-stable noncovalent interaction within the grid^47^. The grid size also indicated the minimal number of side chains of free or silent amino acids that did not participate in any noncovalent interaction within the grid. Uncommon grid sizes were denoted in black numbers on the network map alongside the Grid_s_ with an s-residue size. The total numbers of noncovalent interactions (*N*) and grid sizes (*S*) along the gating pathway of BRTNaC1 from R53 to F414 were calculated and displayed in black and cyan circles, respectively, next to the mesh network map for calculating the systematic thermal instability based on the equation as previously examined^6,12,17,18,22–29^:

1 $${\mathrm{T}}_{{\mathrm{i}}} = S/N$$

### Calculating the melting temperature threshold for heat unfolding

The equation for calculating the melting temperature threshold (T_m,th_) for the heat-induced unfolding of the least-stable noncovalent interaction within a specific grid has been previously examined in several studies^6,12,17,18,22–29^. It is as follows:

2 $${\mathrm{T}}_{{{\mathrm{m}},{\mathrm{th}}}} (^\circ {\mathrm{C}}) = {34} + \left( {{\mathrm{n}} - {2}} \right) \times {1}0 + \left( {{2}0{-}{\mathrm{s}}} \right) \times {2}$$

where, n represents the total number of basic H-bonds (~ 1 kcal/mol each) that are energetically equivalent to the least-stable noncovalent interaction controlled by the given grid, and s is the grid size with the minimal energy required to stabilize the least-stable noncovalent interaction^36^. Therefore, the heat capacity of the grid will increase as the grid size decreases or as the number of equivalent basic H-bonds increases.

For example, without the Y381-D389 H-bond, the Y81-Y381 π bridge in Fig. 2a was controlled by a Grid_10_ due to three free residues from V78 to D80 between H77 and Y81, as well as seven free residues from T382 to S388 between Y381 and D389 in a thermoring from Y81 to H77, D389, Y381 and back to Y81. In this case, when the energy of the Y81-Y381 bridge equaled that of two basic H-bonds (2 kcal/mol), the theoretical T_m,th_ to unfold the Y81-Y381 bridge was calculated to be 64 °C. However, with the presence of the Y381-D389 H-bond, the Y81-Y381 bridge was governed by Grid_3_ because there were only three unbound residues such as V78, L79 and D80 in a thermoring from Y81 to H77, D389, Y381 and back to Y81. In that case, the T_m,th_ for unfolding the Y81-Y381 bridge was calculated to be 68 °C.

### Evaluating the systematic temperature sensitivity

A gating transition of the thermosensitive TRPV1 or TRPV3 channel is always accompanied by a change in the conformational energy density along the lipid-dependent minimal gating pathway^6,12^. Accordingly, for enthalpy-driven activation of BRTNaC1 from a closed state within 10 °C as a result of the broken biggest grid, if the chemical potential of a grid is theoretically defined as the maximal potential for equivalent residues in the grid to form the tightest β-hairpin with the smallest loop via noncovalent interactions^48^, the grid-based structural thermo-sensitivity (Ω_10_) of a single ion channel for heat activation could be defined and calculated using the following equations as examined previously^6,12^.

3 $$\Omega_{{{1}0}} = \left[ {\left( {{\mathrm{S}}_{{\mathrm{c}}} {-}{\mathrm{S}}_{{\mathrm{o}}} } \right){\mathrm{E}}/{2}} \right]^{{({\mathrm{Hc}}/{\mathrm{Ho}})}} = \left[ {\left( {{\mathrm{S}}_{{\mathrm{c}}} {-}{\mathrm{S}}_{{\mathrm{o}}} } \right){\mathrm{E}}/{2}} \right]^{{[({\mathrm{ENc}}/({\mathrm{ENo}})]}} = \left[ {\left( {{\mathrm{S}}_{{\mathrm{c}}} {-}{\mathrm{S}}_{{\mathrm{o}}} } \right){\mathrm{E}}/{2}} \right]^{{({\mathrm{Nc}}/{\mathrm{No}})}}$$

where, along the same gating pathway of one subunit from R53 to F414, N_c_ and N_o_ represent the total noncovalent interactions, H_c_ and H_o_ denote the total enthalpy included in them, and S_c_ and S_o_ indicate the total grid sizes in the closed and open states, respectively. E is the energy intensity of a noncovalent interaction in a range of 0.5–3 kcal/mol. Typically, E is averaged as 1 kcal/mol^36^. This assumption has been validated by matching the experimental functional thermosensitivity of TRPV1 or TRPV3^6,12^. Thus, Ω_10_ factually reflects a thermo-evoked change in the total chemical potential of grids upon a thermo-evoked change in the total enthalpy included in the noncovalent interactions apparently from a closed state to an open state along the same defined gating pathway of one subunit.

For a convenient comparison, the functional thermo-sensitivity (Q_10_) of a single ion channel for heat activation was calculated using the following equation:

4 $${\mathrm{Q}}_{{{1}0}} = \left( {{\mathrm{X}}_{{2}} /{\mathrm{X}}_{{1}} } \right)^{{{1}0/({\mathrm{T2}} - {\mathrm{T1}})}}$$

where, X_1_ and X_2_ are the relative channel activities obtained at temperatures T1 and T2 (measured in Kelvin), respectively.

## Supplementary Information

Below is the link to the electronic supplementary material.

Supplementary Material 1

## Data Availability

All data generated or analysed during this study are included in this published article and supplementary material.

## Abbreviations

ASIC: Acid sensing ion channels
BRTNaC1: Broad-range thermal receptor 1
cryo-EM: Cryogenic electron microscopy
PE: Phosphatidylethanolamine
PC: Phosphatidylcholine
PI: Phosphatidylinositol
PcTx1: Psalmotoxin 1
Ω_10_: Structural thermosensitivity
Q_10_: Functional thermosensitivity
T_i_: Systematic thermal instability
T_m,th_: Melting temperature threshold
TRP: Transient receptor potential
TRPV: TRP vanilloid
TRPVi: (i = 1, 2, 3, 4)
hTRPV1: Human TRPV1
hTRPV3: Human TRPV3
hTRPV4: Human TRPV4
mTRPV3: Mouse TRPV3
rTRPV1: Rat TRPV1
rTRPV2: Rat TRPV2
WT: Wild-type

## References

[1] Caterina, M. J. et al. The capsaicin receptor: A heat-activated ion channel in the pain pathway. *Nature* **389**, 816–824. 10.1038/39807 (1997). 9349813 10.1038/39807

[2] Tominaga, M. et al. The cloned capsaicin receptor integrates multiple pain-producing stimuli. *Neuron* **21**, 531–543. 10.1016/s0896-6273(00)80564-4 (1998). 9768840 10.1016/s0896-6273(00)80564-4

[3] Jordt, S. E., Tominaga, M. & Julius, D. Acid potentiation of the capsaicin receptor determined by a key extracellular site. *Proc. Natl. Acad. Sci. U.S.A.* **97**, 8134–8139. 10.1073/pnas.100129497 (2000). 10859346 10.1073/pnas.100129497 PMC16682

[4] Zhang, K., Julius, D. & Cheng, Y. Structural snapshots of TRPV1 reveal mechanism of polymodal functionality. *Cell* **184**, 5138-5150.e12. 10.1016/j.cell.2021.08.012 (2021). 34496225 10.1016/j.cell.2021.08.012 PMC8488022

[5] Kwon, D. H. et al. Heat-dependent opening of TRPV1 in the presence of capsaicin. *Nat. Struct. Mol. Biol.* **28**, 554–563. 10.1038/s41594-021-00616-3 (2021). 34239123 10.1038/s41594-021-00616-3 PMC8335751

[6] Wang, G. Thermoring-based heat activation switches in the TRPV1 biothermometer. *Int. J. Biol. Macromol.* **248**, 125915. 10.1016/j.ijbiomac.2023.125915 (2023). 37481175 10.1016/j.ijbiomac.2023.125915

[7] Mugo, A. et al. A suicidal mechanism for the exquisite temperature sensitivity of TRPV1. *Proc. Natl. Acad. Sci. U.S.A.* **120**, e2300305120. 10.1073/pnas.2300305120 (2023). 37639609 10.1073/pnas.2300305120 PMC10483596

[8] Deng, Z. et al. Gating of human TRPV3 in a lipid bilayer. *Nat. Struct. Mol. Biol.* **27**, 635–644. 10.1038/s41594-020-0428-2 (2020). 32572252 10.1038/s41594-020-0428-2 PMC7354234

[9] Liu, B. & Qin, F. Single-residue molecular switch for high-temperature dependence of vanilloid receptor TRPV3. *Proc. Natl. Acad. Sci. U.S.A.* **114**, 1589–1594. 10.1073/pnas.1615304114 (2017). 28154143 10.1073/pnas.1615304114 PMC5321025

[10] Shimada, H. et al. The structure of lipid nanodisc-reconstituted TRPV3 reveals the gating mechanism. *Nat. Struct. Mol. Biol.* **27**, 645–652. 10.1038/s41594-020-0439-z (2020). 32572254 10.1038/s41594-020-0439-z

[11] Nadezhdin, K. D. et al. Structural mechanism of heat-induced opening of a temperature-sensitive TRP channel. *Nat. Struct. Mol. Biol.* **28**, 564–572. 10.1038/s41594-021-00615-4 (2021). 34239124 10.1038/s41594-021-00615-4 PMC8283911

[12] Wang, G. Thermoring basis for the TRPV3 bio-thermometer. *Sci. Rep.* **13**, 21594. 10.1038/s41598-023-47100-0 (2023). 38062125 10.1038/s41598-023-47100-0 PMC10703924

[13] Haug, F. M. et al. Functional and structural insights into activation of TRPV2 by weak acids. *EMBO J.* **43**, 2264–2290. 10.1038/s44318-024-00106-4 (2024). 38671253 10.1038/s44318-024-00106-4 PMC11148119

[14] Liu, B. & Qin, F. Use dependence of heat sensitivity of vanilloid receptor TRPV2. *Biophys. J.* **110**, 1523–1537. 10.1016/j.bpj.2016.03.005 (2016). 27074678 10.1016/j.bpj.2016.03.005 PMC4833830

[15] Dosey, T. L. et al. Structures of TRPV2 in distinct conformations provide insight into role of the pore turret. *Nat. Struct. Mol. Biol.* **26**, 40–49. 10.1038/s41594-018-0168-8 (2019). 30598551 10.1038/s41594-018-0168-8 PMC6458597

[16] Mugo, A. N., Chou, R. & Qin, F. Protein dynamics underlies strong temperature dependence of heat receptors. *Proc. Natl. Acad. Sci. U.S.A.* **122**, e2406318121. 10.1073/pnas.2406318121 (2025). 39793069 10.1073/pnas.2406318121 PMC11725839

[17] Wang, G. Pathway-dependent cold activation of heat-responsive TRPV channels. *Res Sq [Preprint]*. rs.3.rs-6450204 (2025). http://oi.org/10.1038/s41598-025-29524-y. Sci Rep (2025). 10.1038/s41598-025-29524-y PMC12749286 41326491

[18] Wang, G. Thermoring basis for thermo-gated TRPV2. *Res Sq [Preprint].* rs.3.rs-6049325. (2025). 10.21203/rs.3.rs-6049325/v2.

[19] Teng, J., Anishkin, A., Kung, C. & Blount, P. Human mutations highlight an intersubunit cation-π bond that stabilizes the closed but not open or inactivated states of TRPV channels. *Proc. Natl. Acad. Sci. U.S.A.* **116**, 9410–9416. 10.1073/pnas.1820673116 (2019). 31010928 10.1073/pnas.1820673116 PMC6511060

[20] Yao, Z. et al. A thermal receptor for nonvisual sunlight detection in myriapods. *Proc. Natl. Acad. Sci. U.S.A.* **120**, e2218948120. 10.1073/pnas.2218948120 (2023). 36780532 10.1073/pnas.2218948120 PMC9974506

[21] Chen, X. et al. Structure and function of a broad-range thermal receptor in myriapods. *Nat. Struct. Mol. Biol.* **32**, 1081–1090. 10.1038/s41594-025-01495-8 (2025). 40011748 10.1038/s41594-025-01495-8

[22] Wang, G. The network basis for the structural thermostability and the functional thermoactivity of aldolase B. *Molecules* **28**, 1850. 10.3390/molecules28041850 (2023). 36838836 10.3390/molecules28041850 PMC9959246

[23] Wang, G. Network basis for the heat-adapted structural thermostability of bacterial class II fructose bisphosphate aldolase. *ACS Omega* **8**, 17731–17739. 10.1021/acsomega.3c00473 (2023). 37251155 10.1021/acsomega.3c00473 PMC10210171

[24] Wang, G. Thermal ring-based heat switches in hyperthermophilic class II bacterial fructose aldolase. *ACS Omega* **8**, 24624–24634. 10.1021/acsomega.3c03001 (2023). 37457467 10.1021/acsomega.3c03001 PMC10339327

[25] Wang, G. Phosphatidylinositol-4,5-biphosphate (PIP_2_)-dependent thermoring basis for cold-sensing of the transient receptor potential melastatin-8 (TRPM8) biothermometer. *Physchem.* **4**, 106–119. 10.3390/physchem4020008 (2024).

[26] Wang, G. Thermoring basis for heat unfolding-induced inactivation in TRPV1. *Nat. Sci.* **4**, e20240008. 10.1002/ntls.20240008 (2024).

[27] Wang, G. ATP-dependent thermoring basis for the heat unfolding of the first nucleotide-binding domain isolated from human CFTR. *Nat. Sci.* **5**, e70007. 10.1002/ntls.70007 (2025).

[28] Wang, G. Trikafta rescues F508del-CFTR by tightening specific phosphorylation-dependent interdomain interactions. *Nat. Sci.* **5**, e70009. 10.1002/ntls.70009 (2025).

[29] Wang, G. Thermodynamic coupling between folding correctors and the first of dimerized nucleotide binding domains in CFTR. *ACS Bio Med. Chem. Au.* **5**, 593–601. 10.1021/acsbiomedchemau.5c00014 (2025). 40860030 10.1021/acsbiomedchemau.5c00014 PMC12371502

[30] Padanilam, B. J. et al. Molecular determinants of intracellular pH modulation of human Kv1.4 N-type inactivation. *Mol. Pharmacol.* **62**, 127–134. https://doi.org/10.1124/mol.62.1.127 (2002). 12065763 10.1124/mol.62.1.127

[31] Watanabe, H. et al. Heat-evoked activation of TRPV4 channels in a HEK293 cell expression system and in native mouse aorta endothelial cells. *J. Biol. Chem.* **277**, 47044–47051. http://doi.org/10.1074/jbc.M208277200 (2002). 12354759 10.1074/jbc.M208277200

[32] Yao, J., Liu, B. & Qin, F. Modular thermal sensors in temperature-gated transient receptor potential (TRP) channels. *Proc. Natl. Acad. Sci. U.S.A.* **108**, 11109–11114. 10.1073/pnas.1105196108 (2011). 21690353 10.1073/pnas.1105196108 PMC3131340

[33] Cui, Y. et al. Selective disruption of high sensitivity heat activation but not capsaicin activation of TRPV1 channels by pore turret mutations. *J. Gen. Physiol.* **139**, 273–283. 10.1085/jgp.201110724 (2012). 22412190 10.1085/jgp.201110724 PMC3315147

[34] Ma, L., Lee, B. H., Clifton, H., Schaefer, S. & Zheng, J. Nicotinic acid is a common regulator of heat-sensing TRPV1-4 ion channels. *Sci. Rep.* **5**, 8906. 10.1038/srep08906 (2015). 25752528 10.1038/srep08906 PMC4894441

[35] Jonstrup, A. T., Fredsøe, J. & Andersen, A. H. DNA hairpins as temperature switches, thermometers and ionic detectors. *Sensors* **13**, 5937–5944. http://doi.org/10.3390/s130505937 (2013). 23666126 10.3390/s130505937 PMC3690039

[36] Neel, A. J., Hilton, M. J., Sigman, M. S. & Toste, F. D. Exploiting non-covalent π interactions for catalyst design. *Nature* **543**, 637–646. http://doi.org/10.1038/nature21701 (2017). 28358089 10.1038/nature21701 PMC5907483

[37] Ryu, S., Liu, B., Yao, J., Fu, Q. & Qin, F. Uncoupling proton activation of vanilloid receptor TRPV1. *J. Neurosci.* **27**, 12797–12807. 10.1523/JNEUROSCI.2324-07.2007 (2007). 18032651 10.1523/JNEUROSCI.2324-07.2007 PMC6673297

[38] Aneiros, E. et al. The biophysical and molecular basis of TRPV1 proton gating. *EMBO J.* **30**, 994–1002. 10.1038/emboj.2011.19 (2011). 21285946 10.1038/emboj.2011.19 PMC3061026

[39] van der Wijst, J. et al. A gate hinge controls the epithelial calcium channel TRPV5. *Sci. Rep.* **7**, 45489. 10.1038/srep45489 (2017). 28374795 10.1038/srep45489 PMC5379628

[40] Dang, S. et al. Structural insight into TRPV5 channel function and modulation. *Proc. Natl. Acad. Sci. U.S.A.* **116**, 8869–8878. 10.1073/pnas.1820323116 (2019). 30975749 10.1073/pnas.1820323116 PMC6500171

[41] Paukert, M. et al. Candidate amino acids involved in H^+^ gating of Acid-sensing ion channel 1a. *J. Biol. Chem.* **283**, 572–581 (2008). 17981796 10.1074/jbc.M706811200

[42] Yoder, N., Yoshioka, C. & Gouaux, E. Gating mechanisms of acid-sensing ion channels. *Nature* **555**, 397–401. 10.1038/nature25782 (2018). 29513651 10.1038/nature25782 PMC5966032

[43] Baconguis, I. & Gouaux, E. Structural plasticity and dynamic selectivity of acid-sensing ion channel-spider toxin complexes. *Nature* **489**, 400–405. 10.1038/nature11375 (2012). 22842900 10.1038/nature11375 PMC3725952

[44] Baconguis, I., Bohlen, C. J., Goehring, A., Julius, D. & Gouaux, E. X-ray structure of acid-sensing ion channel 1-snake toxin complex reveals open state of a Na(+)-selective channel. *Cell* **156**, 717–729. 10.1016/j.cell.2014.01.011 (2014). 24507937 10.1016/j.cell.2014.01.011 PMC4190031

[45] Gonzales, E. B., Kawate, T. & Gouaux, E. Pore architecture and ion sites in acid-sensing ion channels and P2X receptors. *Nature* **460**, 599–604. 10.1038/nature08218.doi:10.1074/jbc.M706811200 (2009). 19641589 10.1038/nature08218 PMC2845979

[46] Cahill, J., Hartfield, K.A., Heusser, S.A., Poulsen, M.H., Yoshioka, C., Pless, S.A., & Baconguis, I. Conformational plasticity of human acid-sensing ion channel 1a. *bioRxiv* [Preprint]. 2024.12.11.628012 (2024). 10.1101/2024.12.11.628012. 10.1038/s41594-026-01845-0 PMC13468109 42463865

[47] Floyd, R. W. Algorithm-97. *Shortest Path. Commun. Acm.* **5**, 345–345 (1962).

[48] Kiehna, S. E. & Waters, M. L. Sequence dependence of β-hairpin structure: Comparison of a salt bridge and an aromatic interaction. *Protein Sci.* **12**, 2657–2667. 10.1110/ps.03215403 (2003). 14627727 10.1110/ps.03215403 PMC2366975

