# Supplementary material for: Thermoring basis for the proton-driven heat activation of a cation-selective channel in myriapods
Source: Sci Rep. 2025 Dec 15;16:1949. doi: 10.1038/s41598-025-31732-5 (PMC12804983; doi:10.1038/s41598-025-31732-5)
Supplement: Supplementary file 1 — Supplementary Material 1 [file 41598_2025_31732_MOESM1_ESM.pdf]

Supporting Information for

Thermoring basis for the proton-driven heat activation of a cation-selective channel in myriapods

Guangyu Wang 1, 2*

[1]Department of Physiology and Membrane Biology, University of California School of Medicine, Davis, CA, USA

[2]Department of Drug Research and Development, Institute of Biophysical Medico-chemistry, Reno, NV, USA

* Correspondence: gary.wang10@gmail.com

This supplementary material includes:

Tables S1, S2, S3, S4 and S5.

Table S1. Tertiary noncovalent interactions along the gating pathway from R53 to F414 in each subunit of closed BRTNaC1 at pH 8 and 4 °C (PDB ID, 8YMR).

Noncovalent interaction	Cut-off distance	Linked residues
Salt bridge	3.2-4 Å	K129-D132, E161/D172-R195, R207-E362, D217-R220, R238-E284, R244-E332, E284/E288-R305, K340-E343
H-bond	<3.9 Å donor-H-acceptor <60°	S70-F397, D80-S83, E84-R268, R99-E230/E342, E102-N190, T104-H229, F110-N113, D132-R318, W145-S148, N146-R149, S152-D210/N154, D172-N174, T184-R238/Y239, R195-P196, Y199-A368, D210-Q212, R214-S360, D215-R220, R259-E349, Q262-N264, V274-R339/C341, D285-R305, R290-E293, R300-P309/C311, Y303-T324, K333-E334, E342-N344
π–π interaction	2.65–6.5 Å	Y81-Y381, F110-F116/F130/W145/F321, F116-F130-F321, W145-Y303/F321, H160-F205, F205-Y366, Y227-H229, F228-F256, H231-H232, H233-H235-Y279, Y246/F254/F256-F350, Y246/F254-F256, F260-Y346, Y369-Y371
cation–π interaction	<6.0 Å	Y170-R200, Y265-R339
CH_3/CH–π interaction	2.65-3.01 Å	W59-V63, F60-V64, I69-F397, H77-D389, A94-Y371, I100-F367, P101-Y346, L133-Y139, W145-P302, Y199-N370, Y227-R244, F228-I258, Y292-R305, F331-E332
Lone pair–π interaction	3-3.7 Å	F286-S335, M351-Y366

Table S2. Tertiary noncovalent interactions along the gating pathway from R56 to V411 in each subunit of closed BRTNaC1/D217N/E218Q at pH 7 and 4 °C (PDB ID, 8YMW).

Noncovalent interaction	Cut-off distance	Linked residues
Salt bridge	3.2-4 Å	R89-D91, E161/**D172-R195**, **R238-E284**, R244-E332, R294-D326
H-bond	<3.9 Å donor-H-acceptor <60°	Q95-N370, E102-H229, T104-S237, R108-Q112, I109-N113-T117, **Q123-R136**-Y139, D132-R318, N146-Y182/Q187, S152-D210, E161-R200, S176-P192, T184-Y239, Y199-A368, G201-T204, R205-E288, R207-D362, N215-R220, **Y227-R244**, E230-T344, S237-T241,Y239-E288, R259-E349, Q262-N264, D285-R305, R290-E293-Q296, Y292-C299, R300-M304, D326-N330, S357-I359, Y381-D389
π–π interaction	2.65–6.5 Å	Y81-Y381, **F110-F116**/F130/**W145**/F321, F116-F130, F130/W145-F321, **H160-F205**, **F205-Y366**, **Y227-H229**, **H233-H235-Y279**, Y246/**F254**/F256-**F350**, Y246/F254-F256, Y303-F321, **Y369-Y371**
cation–π interaction	<6.0 Å	**Y265-R339**, Y279-K340
CH_3/CH–π interaction	2.65-3.01 Å	V60-F64, I69-F397, H77-D389, **A94-Y371**, I100-F367, **P101-Y346**, L133-Y139, W145-P302, **Y199-N370**, E230-H233, V248-F254, **I263-Y265**
Lone pair–π interaction	3-3.7 Å	F286-S335, M351-Y366

Note: Bold interactions were conserved in closed and open states.

Table S3. Tertiary noncovalent interactions along the gating pathway V63 to I400 in each subunit of open BRTNaC1/D217N/E218Q at pH 7 and 40 °C (PDB ID, 8YMX).

Noncovalent interaction	Cut-off distance	Linked residues
Salt bridge	3.2-4 Å	D118-K122, **D172-R195**, **R238-E284**, R259-E349, E288-R300
H-bond	<3.9 Å donor-H-acceptor <60°	R89-D91, I100-H231, E102-N190, P107-S185, F110/Q111-T117, **Q123-R136**, D140/I141-S312, N146-R149, S148-Q187, S151-Q187, E161-R195/I198, T183-S237, T184-R238/D325, N215-N217, **Y227-R244**, H233-S237, H235-R238/A280, R244-E332, S257-E349, R259/F260-Q373, Y265-E343, D275-Y279, E278-K338, Q289-R290, E293-K294, S312-R318
π–π interaction	2.65–6.5 Å	F92-Y369, **F110-F116/W145**, W145-Y182, **H160-F205**, **F205-Y366**, **Y227-H229**, **H233-H235-Y279, F254-**F256/**F350**, F260-Y346, Y292-F321, F350-H352, **Y369-Y371**
cation–π interaction	<6.0 Å	Y170-R200, **Y265-R339**
CH_3/CH–π interaction	2.65-3.01 Å	V63-F65**, A94-Y371**, **P101-Y346**, **E102-H229**, F135-T315, **Y199-N370**, Y199-T204, H235-V283, F256-M363, **I263-Y265,** Y279-K340, V297/R300-F321, F331-E332
Lone pair–π interaction	3-3.7 Å	N113-F116

Note: Bold interactions in BRTNaC1/D217N/E218Q were conserved upon heat activation from 4 to 40 °C.

Table S4. Tertiary noncovalent interactions along the gating pathway from R53 to F414 in each subunit of closed BRTNaC1 at pH 4 and 4 °C (PDB ID, 8YMS).

Noncovalent interaction	Cut-off distance	Linked residues
Salt bridge	3.2-4 Å	**K122-E218**, E132-R318, E161-R195/R200, **D172-R195**, **R244-E332**, **E284-R305**, R290-E334, R339-E343
H-bond	<3.9 Å donor-H-acceptor <60°	C66-S70-V393, N113-I115, D126-M128/K129, **R136-Y139**, N146/S148-Q187, **R149-F151**, I150-Q212, **C157-Q177**, S158-H160, P173/M175-Q177, **T184-R238**, Y199-A368, T204-F367, Q212-S360, P213-R220, R214-P221, **Q225-G245**, **Q262-N264-T376**, R268-E377, **D285**/E288-**R305**, Y303-T324, F331-E334, S404-E407
π–π interaction	2.65–6.5 Å	**Y81-Y381**, **F110-F116/F130/W145/F321**, **F116-F130-F321**, **W145-F321/Y303**, **H160-F205**, **F205-Y366**, **Y227-H229**, **F228-F256**,**Y239-Y303**, **Y246/**F254/**F256-F350**, **Y246/F254-F256**, **F260-Y346**, Y303-Y321
cation–π interaction	<6.0 Å	R89-Y378, Y265-K338
CH_3/CH–π interaction	2.65-3.01 Å	F60-V64, I69-F397, H77-D389, **A94-Y371**, I100-F367, **P101-Y346**, **L133-Y139**, **W145-P302**, T184-Y303, **Y199-N370**, F228-I258, F331-E332
Lone pair–π interaction	3-3.7 Å	**D80-Y381**, E138-Y139, M351-Y366

Note: Bold interactions in BRTNaC1 were conserved in the closed and open states at pH 4 and 4 °C.

Table S5. Tertiary noncovalent interactions along the gating pathway from R53 to F414 in each subunit of open BRTNaC1 at pH 4 and 4 °C (PDB ID, 8YMU).

Noncovalent interaction	Cut-off distance	Linked residues
Salt bridge	3.2-4 Å	R108-E222, **K122-E218**, **D172-R195**, **R244-E332**, R259-E349, R268-E377, **E284/E288-R305**
H-bond	<3.9 Å donor-H-acceptor <60°	Q73-H77-Y381, Q95-E97/N370, R99-S194/R195, E102-N190, T104-S237, D126-K129, D132-R318, **R136-Y139**, **R149-F151**, S152-V155, **C157-Q177**, G162-S169, **T184-R238**, T184-Y239, **Y199-A368**, R207-D362, **Q212-S360**, **Q225-G245**, H229-S237, E230-N344, H232-E277, H235-Y239-E288, **Q262-N264-T376**, A266-E377, **D285-R305**, R290-E293, K294-D326, R300-L308/P309, I320-T324, D325-Q329
π–π interaction	2.65–6.5 Å	**Y81-Y381**, **F110-F116/F130/W145/F321**, **F116-F130-F321**, **W145-F321/Y303**, **H160-F205**, **F205-Y366**, **Y227-H229**, F228-Y246, **F228-F256**, **Y239-Y303**, **Y246/F256-F350**, **Y246/F254-F256**, **F260-Y346**
cation–π interaction	<6.0 Å	Y265-R339
CH_3/CH–π interaction	2.65-3.01 Å	V63-F67, **A94-Y371**, **P101-Y346**, **L133-Y139**, M142-Y182, **W145-P302**, F151-I189, **Y199-N370**, F286-S335
Lone pair–π interaction	3-3.7 Å	**D80-Y381**

Note: Bold interactions in BRTNaC1 were conserved in the closed and open states at pH 4 and 4 °C.
